# Comparison of the Thresholds of Households’ Exposure to Catastrophic Health Expenditure in Iran and Brazil, and Selection of the Most Appropriate Threshold

**Published:** 2018-12

**Authors:** Arash RASHIDIAN, Ali AKBARI SARI, Seyed Mostafa HOSEINI, Moslem SOOFI, Hoseyn AMERI

**Affiliations:** 1. Dept. of Health Management and Economics, School of Public Health, Tehran University of Medical Sciences, Tehran, Iran; 2. Dept. of Epidemiology and Biostatistics, School of Public Health, Tehran University of Medical Sciences, Tehran, Iran

**Keywords:** Catastrophic health expenditure, Receiver operating characteristic curve, Kappa coefficient

## Abstract

**Background:**

Different definitions are nowadays adopted to estimate the threshold of exposure of households to catastrophic health expenditures and different thresholds are used in various studies. This study was conducted to compare these thresholds and select the most appropriate threshold for defining catastrophic health expenditure in Iran and Brazil.

**Methods:**

In this cross-sectional study, data were collected from 592 households from District 17 of Tehran, Iran, and 869 households from Porto Alegre, Brazil in 2013. Firstly, catastrophic health expenditures were calculated using two common proportions, i.e. out-of-pocket health payments, as a proportion of total cost and as a proportion of ability to pay. These proportions were analysed using the ROC curve and Kappa coefficient.

**Results:**

The appropriate cut off point for the thresholds of 5%, 10%, 15%, and 20% of the total expenditure was 0.52, 0.34, 0.28, and 0.46 in Iran, and 0.44, 0.36, 0.28, and 0.23 in Brazil, respectively. The appropriate cut off point for the thresholds of 20%, 25%, 30%, 35%, and 40% of ability to pay was 0.31, 0.28, 0.25, 0.34, and 0.40 in Iran and 0.36, 0.34, 0.30, 0.38, and 0.46 in Brazil, respectively.

**Conclusion:**

The appropriate cut off point for the proportion of out-of-pocket health payments to total expenditure and proportion of health expenditure to ability to pay was the threshold of 20% of total expenditure and 35% of ability to pay, respectively.

## Introduction

Catastrophic health expenditure has been defined as “payment for receiving health services when exceeding a certain proportion of the total household expenditure” (1–2). However, the question here is about defining the “certain proportion” indicating the exposure of the household to catastrophic health expenditure. Most researchers have often used two approaches to estimate this proportion in the definition of catastrophic health expenditure. In the old approach, catastrophic health expenditure was defined as expenditure that exceeded a certain proportion of the income and total expenditure of the household in a certain period of time, for example in one year (3–5). Spending a major proportion of the household’s income on health services may keep the household from using other products and services which might be vital and essential to the survival of the household in the short term, and may result in selling of household properties, debt, spending household savings, and experiencing economic hardship in the long term (6–8). However, when total expenditure of the household is regarded as the denominator, catastrophic health expenditures are defined relative to the health payments budget share. A potential problem is that poor households in low-income countries may have a low budget share. The severity of the budget means that most of the resources of the household are spent on essential products and services like food, clothing, education, housing, etc. and the remainder of the budget is spent on health services. Therefore, the households that cannot pay for health services are not taken into account in this definition (9, 10).

A new approach was presented in which catastrophic health expenditures are regarded as a share of ability to pay. ‘Household’s capacity to pay’ is calculated through subtracting expenditures on basic needs from the total expenditure or total income of the household (11). Although the new approach removes the problems of the old one, to some extent, the main question regarding the threshold of catastrophic health expenditure remains unanswered. Any threshold was inevitably a matter of choice, and a range of 2.5%–15% of total expenditure and 10%–40% of ability to pay could be chosen for use in defining catastrophic health expenditure (12).

A threshold of 40% of ability was proposed to pay for the definition of catastrophic health expenditure in a study of 59 countries in 2003, there is more agreement on such threshold (11–13). Similarly, the threshold of 40% of ability to pay is also suggested by WHO (14) although the World Bank regards a threshold of 20% of total income for the exposure of households with catastrophic health expenditure (15). Considering the aforementioned, different researchers have used different thresholds for the definition of catastrophic health expenditure; therefore, we decided to perform this study to compare and select an appropriate threshold using the ROC curve and Kappa coefficient. Finding appropriate threshold of exposure to catastrophic health expenditures provide better understanding of such expenditures and, therefore, can help policymaker in designing more effective policies for protecting people against financial burden of health cost.

This study was conducted to compare thresholds and to select the most appropriate threshold for defining catastrophic health expenditure.

## Materials and Methods

### Data collection

In 2013, we asked several countries such as Brazil, Georgia, Turkey, Thailand and so forth. None of them were willing to provide the respective data, except Brazil and Iran. Therefore, the present descriptive analytical study was performed in the households of District 17 of Tehran, Iran (16) and Porto Alegre, Brazil (17). Data of studies performed in District 17 of Tehran and Porto Alegre, Brazil, were used, the study population was the sum of the study populations of the two studies, i.e. 1461 households (869 households from Porto Alegre and 592 households from District 17 of Tehran). The study was approved by Ethical Committee of Tehran University of Medical Sciences.

### Instrument

In Iran, the socioeconomic data of the households was collected through a questionnaire designed by the WHO entitled “World Health Survey”. This questionnaire had been developed in 2003 to assess the performance of the health systems in terms of responsiveness, delivering services and financial contribution (18). The validity and reliability of translated questionnaire were determined and verified (16). In Brazil, a standardized, pre-coded questionnaire had used for the collecting data. Data quality control carried out and resolved all discrepancies using Epi-Info 6.04. (19). We used the same variables in two dataset to conduct the study. Analysis of the different thresholds was performed in three stages.

### Statistical analysis

#### First stage

Catastrophic health expenditures were calculated through $\frac{\text{T}}{\text{X}}$ [1] and $\frac{T}{X-\text{f}(\text{x})}$ [2] equations representing the old and new approach respectively, where T shows out of pocket payments (OOP) (the costs of medications, dental services, diagnosis, outpatient visits, hospitalization, and traditional healers), and X, f(x) and X-f(x) represent total expenditure (TE) of the household, substitute expenditure (SE) of the household, and household’s ability to pay, respectively (20). To calculate the household’s ability to payand substitute expenditure we used the method (11) in which ability to pay is the difference between total household expenditures and subsistence expenditure. Subsistence expenditure was calculated as the average food expenditures of the households whose food share fall within the 45^th^ to 55^th^ percentile range across the whole sample, however adjusted based on size of household (16, 21).

#### Second stage

The households driven below the poverty line due to health expenditure were calculated. To identify the number of these households, out of pocket payments were subtracted from total expenditure. If the result was less than the substitute expenditure of the household, the household was driven below the poverty line. Poor =1 if TE-OOP<SE [3]

While identifying the households driven below the poverty line, the households which were already below the line of poverty before paying health expenditure were excluded from calculations. To identify these households, substitute expenditure was compared with total expenditure of the household. If substitute expenditure was more, the household was below the line of poverty and excluded from the study (11, 22).

#### Third stage

To determine the appropriate cut off point, the data of the two aforementioned equations were analyzed and the number of households driven below the line of poverty due to health expenditure, regarded as the gold standard in this study, was calculated. To measure the compatibility of the thresholds in the two approaches with the number of households driven below the line of poverty, Kappa coefficient, calculated using the following formula, was employed (Table 1). Kappa coefficient ranges from 0 to 100 and values close to 100 indicate appropriateness. Stata version 11 was used for data analysis.

**Table 1:** Kappa formula

***Parameter***	***Non-exposed***	***Exposed***	***Driven below poverty line***
A+B	B	A	**+**
C+D	D	C	−

$$\mathrm{Kappa}\text{=}\frac{\frac{A+B}{N}-\left(\frac{\left(A+B)(A+C\right)}{N}+\frac{\left(C+D)(B+D\right)}{N}\right)}{1-\left(\frac{\left(A+B)(A+C\right)}{N}+\frac{\left(C+D)(B+D\right)}{N}\right)}$$			

## Results

The socioeconomic data of 1461 households were included in the study. Table 2 presents the characteristics of the households based on the variables of age, household size, insurance status, and economic status. The mean age of the participants was 44.9 yr in Brazil and 47.5 yr in Iran. Regarding the size of the households, most of the households had 3–7 members in both studies. The mean household size was 3.84 persons in Iran and 3.55 in Brazil. The mean number of the individuals with health insurance was 65.4% in Brazil and 73.6% in Iran. Due to the nature data available in each survey, the economic quintiles in Iran were based on the total expenditure of the household and on Brazil they were identified based on the total income of the households.

**Table 2:** The descriptive analysis of the households based on demographic variables

***Variable Age(yr)***	***Brazil Perce***	***Iran Percent***
≤5	13.55	5.38
6–14	17.74	11.75
15–34	33.83	43.93
35–64	29.51	32.01
65≥	5.39	6.93
**Size of the household**		
≤2	4.2	30.05
3–6	78.2	62.64
7≥	18.6	7.31
**Medical insurance**		
Positive	73.6	77.8
Negative	26.4	22.2
**Socioeconomic status**		
1^st^ quintile	21.6	37.49
2^nd^ quintile	10.8	26.08
3^rd^ quintile	28.1	19.91
4^th^ quintile	15.2	11.98
5^th^ quintile	24.3	4.54

Table 3 demonstrates the frequency of the households’ exposure to catastrophic health expenditure based on both approaches. The highest exposure was related to the thresholds of 5% of total expenditure and 20% of ability to pay in both countries. The rate of the households’ exposure to catastrophic health expenditure decreased with increase of the thresholds in both countries. Table 4 presents Kappa coefficient between the households identified as facing catastrophic health expenditure at different thresholds with the households driven below the poverty line because of health care expenditure. The Kappa coefficient were around 95% where the threshold of 45% of ability to pay was used in both countries (Kappa=95.2% and 94.6%; for Brazil and Iran, respectively); and it was around 90% where the threshold of 25% of total expenditure was utilized (89.0% and 90.2%; for Brazil and Iran, respectively).

**Table 3:** The frequency of the households’ exposure to catastrophic health expenditure at different thresholds

***Country***	***OOP health spending exceeding X% of total expenditure***					***OOP health spending exceeding X% of the ability to pay***				
	***5%***	***10%***	***15%***	***20%***	***25%***	***20%***	***25%***	***30%***	***35%***	***40%***
Iran	54.6	35.8	21.8	16.6	11.1	28.7	21.8	17.2	12.5	11.8
Brazil	46.5	28.8	20.1	16.1	13.4	21.3	18.2	15.8	13.2	12.0

**Table 4:** Kappa coefiicients between different threshold levels of total expenditure or ability to pay and the proportion of households driven below the poverty line

***Country***	***OOP health spending exceeding X% of total expenditure***					***OOP health spending exceeding X% of the ability to pay***				
	***5%***	***10%***	***15%***	***20%***	***25%***	***20%***	***25%***	***30%***	***35%***	***40%***
Iran	50.2%	68.2%	81.3%	85.8%	90.2%	75.0	86.2	87.1	91.9	94.6
Brazil	56.9%	74.3%	82.9%	86.3%	89.0%	82.1	87.5	88.2	92.3	95.2

**Fig. 1:**
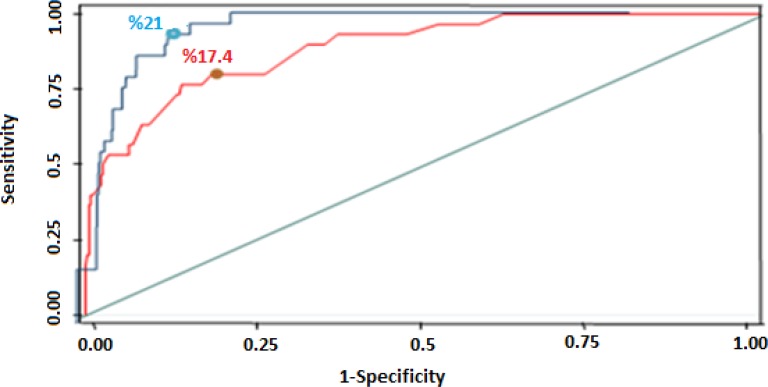
The ROC curve for the 1-specificity and sensitivity of the proportion of OOP health payment a total expenditure as compared with the proportion of household driven below the poverty line

Fig. 1 shows the ROC curve for the thresholds of ratio of health expenditure to total expenditure of the household and the number of households driven below the poverty line. The area under the ROC curve was 0.88 and 0.93 for the studies in Iran and Brazil respectively, indicating the appropriateness of the curve.

For Iran, the cut off points for the thresholds of 5%, 10%, 15%, 20%, and 25% of total expenditure were 0.52, 0.34, 0.30, 0.28, and 0.46, respectively. The lowest and the best cut off point was 0.27 related to the threshold of 17.4% of total expenditure. For Brazil, the cut off points for similar thresholds were 0.44, 0.36, 0.28, 0.23 and 0.31, respectively; the lowest cut off point was 0.17 related to the threshold of 21% of total expenditure.

**Fig. 2:**
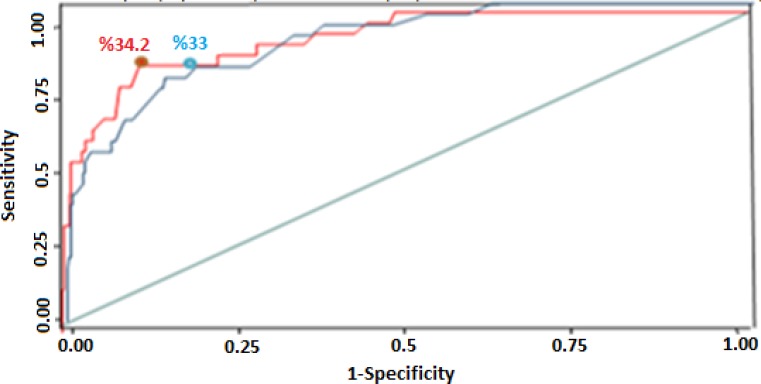
The ROC curve for the 1-specificity and sensitivity of the proportion of OOP health payment an ability to pay as compared with the proportion of household driven below the poverty line

Fig. 2 shows the ROC curve for the thresholds of the proportion of health expenditure to ability to pay and the number of households driven below the poverty line. The area under the ROC curve was 0.91 and 0.90 for the studies in Iran and Brazil respectively, indicating the appropriateness of the curve.

In the Iranian study, the cut off points for the thresholds of 20%, 25%, 30%, 35%, and 40% of the ability to pay were 0.31, 0.28, 0.25, 0.34, and 0.40, respectively. The lowest and the best cut off point was 0.20 related to the threshold of 33% of the households’ ability to pay. In the Brazilian study, the cut-off points for similar thresholds were 0.36, 0.34, 0.30, 0.38, and 0.46, respectively. The lowest cut off point was 0.27 related to the threshold of 34.2% of households’ ability to pay.

## Discussion

The aim of the study was to compare the thresholds of households’ exposure to catastrophic health expenditure, and selection of the most appropriate threshold. The difference between the thresholds obtained in this study and the threshold proposed by the WHO and the World Bank is insignificant.

Among the thresholds of the ratio of out-of-pocket health payments to total expenditure, a threshold of 20% of total expenditure was a more appropriate method for measuring catastrophic health expenditure, since the appropriate cut off point among the thresholds was 17.4% with a sensitivity of 77.4% and a specificity of 86.4%. In other words, this threshold better identified the households driven below the line of poverty. For the Brazilian study, the threshold was 21% with a sensitivity of 80.1% and a specificity of 84.1%. However, the appropriate cut off points of the threshold of 20% and the two cut off points of the thresholds of 17.4% and 21% were not very different.

This finding is similar to cut-off of 20% of all expenditures suggested by the World Bank (15).

The appropriate cut off point among the thresholds of the ratio of out-of-pocket health payments to ability to pay was the threshold of 35%. The thresholds from the Iran and Brazil studies, as appropriate cut off points, were in the range of 30%–40% of ability to pay. In addition, reanalysis considering the thresholds of 25%, 30%, 35%, 40%, and 45% of ability to pay showed that the best cut off point was the threshold of 35%. According to ROC curve analysis the appropriate cut-off points in term of capacity to pay in the range of 30%–40% were 33% and 34.2% for Brazil and Iran, respectively. Therefore, we take 35 into account as appropriate cut-off point.

The results of Kappa coefficient showed that the thresholds of 20% of total expenditure and 35% of ability to pay presented a better definition of catastrophic health expenditures. In both studies, Kappa increased with increase of the thresholds to 20% of total expenditure and 35% of ability to pay and decreased thereafter, indicating that the majority of the households driven below the line of poverty were detected in the aforementioned thresholds.

To the best of our knowledge, this is the first study using the ROC and Kappa methods to comparison of the thresholds for better definition of CHE. It can be a benchmark for next case studies. Our study has also own limitations. Our results was obtained from data on Iran and Brazil so the finding should be interpreted by cautious. Because of relatively small sample it is difficult to reach a conclusion, thus, a large scale study may need to be conducted for better conclusion. Moreover, since no study has evaluated and compared the methods of measuring catastrophic health expenses to date, we only interpreted the results of our study.

## Conclusion

Since Kappa coefficient’s effectiveness in detecting the households driven below the poverty line due to catastrophic health expenditure between the thresholds of 20% of total expenditure and 35% of ability to pay was 95.6 and 91.0 in the Iran and Brazil studies, and because poor households allocate a higher share of their total expenditure to food and ignore health expenses, considering a threshold of 20% of total expenditure does not include the poor households in the households exposed to catastrophic health expenses. Therefore, using a threshold of 35% of ability to pay is a better method for measuring catastrophic health expenses.

## Ethical considerations

Ethical issues (Including plagiarism, informed consent, misconduct, data fabrication and/or falsification, double publication and/or submission, redundancy, etc.) have been completely observed by the authors.
